# Angioedema and COVID-19: A New Dermatological Manifestation?

**DOI:** 10.3390/idr13010004

**Published:** 2021-01-01

**Authors:** Pierre-Yves Royer, Souheil Zayet, Claire Jacquin-Porretaz, N’dri Juliette Kadiane-Oussou, Lynda Toko, Vincent Gendrin, Timothée Klopfenstein

**Affiliations:** 1 Infectious Disease Department, Nord Franche-Comté Hospital, 90400 Trevenans, France; souheil.zayet@hnfc.fr (S.Z.); kadianeoussou14@gmail.com (N.J.K.-O.); lynda.toko@hnfc.fr (L.T.); vincent.gendrin@hnfc.fr (V.G.); timothee.klopfenstein@hnfc.fr (T.K.); 2 Dermatology Department, Nord Franche-Comté Hospital, 90400 Trévenans, France; claire.jacquinporretaz@hnfc.fr

**Keywords:** COVID-19, angioedema, dermatology

## Abstract

The main localization of SARS-CoV-2 infection is the respiratory tract. Digestive and otorhinolaryngological localizations are also reported. More recently, dermatological manifestations have been reported during Coronavirus disease-19 (COVID-19). We report a case of a labial angioedema in a patient with confirmed COVID-19.

## 1. Introduction

The clinical expression of coronavirus disease 2019 (COVID-19) is polymorphic. The respiratory tract is mainly involved in acute respiratory distress syndrome (ARDS). Digestive and otorhinolaryngological symptoms are also described [1,2]. Currently, there are a few dermatological features described [3,4] but no angioedema. We report a case of a labial angioedema in a patient with a confirmed case of COVID-19 proved by RT-PCR. 

## 2. Case Report

**Figure 1:**
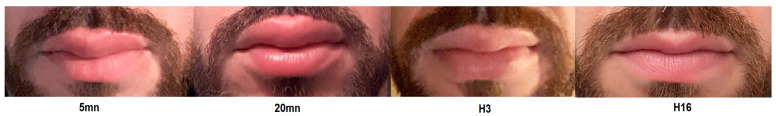
Labial angioedema evolution during COVID-19, from 5 mn to H16.

On 24 March, a 34-year-old male presented a labial angioedema. He was not taking any medication and he had no comorbidities except asthmatic manifestations to grass pollens and cat hair. He presented, on 17 March, retro-orbital headache and rhinorrhea. On 20 March, he described anosmia and ageusia, without nasal congestion. He did not present respiratory or digestive symptoms. Therefore, chest radiography was not carried out, nor was blood examination, because of the good general state of the patient. Severe acute respiratory syndrome coronavirus 2 (SARS-CoV-2) RT-PCR of the nasopharyngeal sample was positive with a viral load of 7.4 log copies/mL. Sensation of fever and asthenia appeared 6 days after the first symptoms. After 7 days, he suddenly presented a labial limited angioedema, without pruritus and erythema. This symptom persisted for three hours and progressively disappeared. He completely recovered after sixteen hours (Figure 1). 

No medication or allergic trigger factors were found. The flu-like syndrome (fever, asthenia, headache and myalgia) of COVID-19 disappeared on the 28th of March. However, after 26 days of follow up, partial anosmia and ageusia persisted.

## 3. Discussion

Angioedema is considered a deep urticaria. It is a hypodermic edema, affecting the skin or mucosa, and is not systematically associated with superficial urticaria [5]. The pathogenesis is explained by capillary vasodilatation with augmentation of permeability due to inflammatory mediators, such as cytokines [5]. Angioedema essentially affects the eyelids and the lips when it is localized on the face. Acute urticaria has been described in infectious diseases, in particular with infections caused by herpes simplex virus (HSV), cytomegalovirus (CMV) and Epstein–Barr virus (EBV) [6]. Urticaria is described with COVID-19 [7,8]. Our case presents a deep urticaria with COVID-19. To our knowledge, there has not been any description in the medical literature of angioedema and SARS-CoV-2 infection to date. The dermatological manifestations mainly associated with COVID-19 are maculopapular rash or frostbite-like lesions [9]. Currently, the French Society of Dermatology is compiling these manifestations for further exploration. In our case patient, angioedema is likely associated with COVID-19. Indeed, this manifestation appeared during the second week of the onset of the disease, corresponding to a major inflammatory stage with severe cytokine storm [10,11]. Angioedema could be explained by inflammatory cascade and cytokine discharge. In *Nord Franche-Comté Hospital*, between 1 March and 14 March, 68 patients were diagnosed with COVID-19, without any urticarial symptoms; the average load was 5.5 log copies/mL for these 68 patients versus 7.4 log copies/mL for our patient. The highest viral load could partly explain an enhanced inflammatory state in our patient. Furthermore, urticaria in COVID-19 could occur most frequently in patients with favorable factors, such as allergic conditions—such was the case for our patient. 

## 4. Conclusions

COVID-19 symptoms seem to be polymorphic, and the skin is not exempt. During the COVID-19 outbreak, we must discuss this diagnostic in the presence of flu-like syndrome and/or the notion of epidemiological exposition and atypical dermatologic symptoms. Further data are needed to confirm the association between COVID-19 and angioedema.

## Data Availability

Data available on request due to privacy restrictions. The data presented in this case study are available on request from the corresponding author.
